# Sweat-Resistant Parylene-C Encapsulated Conductive Textiles for Active Thermal Management

**DOI:** 10.3390/polym17212952

**Published:** 2025-11-05

**Authors:** Shi Hu, Dan Wang, Mohanapriya Venkataraman, Jiří Militký, Dana Křemenáková, Martin Palušák

**Affiliations:** 1 Department of Material Engineering, Faculty of Textile Engineering, Technical University of Liberec, Studenská 1402/2, 46117 Liberec, Czech Republic; shi.hu@tul.cz (S.H.); dan.wang@tul.cz (D.W.);; 2 Department of Environmental Chemistry, The Institute for Nanomaterials, Advanced Technologies and Innovation, Technical University of Liberec, Studentská 1402/2, 46001 Liberec, Czech Republic

**Keywords:** Parylene-C encapsulation, copper-coated textile, electro-thermal heating, sweat resistance, chemical vapor deposition (CVD), thermal insulation, smart wearable fabrics

## Abstract

The development of electro-thermal textiles has attracted growing interest as a promising approach for active thermal management in wearable systems. Metallic-coated fabrics can efficiently generate heat through the Joule effect; however, their long-term performance and safety are severely limited under perspiration due to metal ion release and corrosion. To overcome these challenges, this study introduces a Parylene-C encapsulation strategy for copper-coated polyethylene terephthalate nonwovens (CuPET) using a chemical vapor deposition (CVD) process. The conformal, biocompatible Parylene-C films (thickness 4–16 μm) act as effective protective barriers while preserving the porous textile structure. Morphological and comfort analyses demonstrate a controlled reduction in air permeability from 3100 to 1100 L·m^−2^·s^−1^, maintaining acceptable breathability. Electro-thermal measurements reveal rapid and uniform heating, reaching 40–45 °C within 2 min at 2 V, and the addition of a thermal insulation layer further enhances the Joule heating efficiency, increasing the steady-state temperature by approximately 6 °C. ICP–OES results show an ≈80% reduction in copper ion release (from 28.34 mg·L^−1^ to 5.80 mg·L^−1^) after artificial sweat exposure. This work demonstrates a scalable encapsulation route that effectively balances sweat protection, electrical stability, and thermal performance, paving the way for safe, durable, and actively heated smart textiles for advanced thermal insulation applications.

## 1. Introduction

With the rapid development of wearable electronics and functional fabrics, smart textiles have evolved from passive thermal insulators to active thermal management systems capable of responding to environmental and physiological stimuli [1,2,3,4]. Among these, electro-thermal textiles, which convert electrical energy into heat through the Joule heating effect, have attracted increasing attention for applications in personal thermal regulation, protective clothing, and adaptive insulation systems [5,6,7]. The integration of metallic conductors—such as silver, copper, nickel, or stainless steel—into textile architectures enables efficient heat generation upon low-voltage activation, providing a promising pathway for self-heating garments in cold environments [8,9,10,11].

However, one of the most critical challenges for metallic or metallized fabrics lies in their chemical instability and biocompatibility under perspiration conditions [12,13,14]. Human sweat is a complex electrolyte containing water, chloride ions, lactate, and urea, which can promote metal oxidation, corrosion, and ion release [15,16,17,18]. Prolonged contact between sweat and metallic surfaces may lead to significant degradation of electrical conductivity and, more importantly, metal ion leaching into the environment or human skin [19,20]. Excessive exposure to metal ions such as Cu^2+^, Ni^2+^, or Ag^+^ has been linked to skin irritation, allergic reactions, and potential heavy metal accumulation [21,22,23,24]. In addition, uncontrolled copper ion release may raise environmental concerns regarding heavy metal contamination [25]. Despite these issues, most reported electro-thermal textiles lack efficient protective strategies to mitigate the corrosive effects of sweat, particularly during long-term wear or high-humidity operation [26,27,28,29].

To address these challenges, surface encapsulation and barrier coatings have been investigated as effective means to enhance the chemical stability of conductive textiles. For example, Zhu et al. coated silver nanowire networks with a polyurethane (PU) layer, achieving improved oxidation resistance [30]; Qi-Wei Wang et al. [31] reported that silicon-coated MXene-decorated polyester textiles exhibited excellent electrical conductivity. Moreover, the electrical conductivity remained stable even after multiple washing cycles with a commercial detergent solution. Di Xing et al. [32] developed a PU film-covered carbon fabric with plated silver (Ag) particles, achieving high electrical conductivity. However, the air permeability and washability of this material were not evaluated. Lihua Zou et al. reported that a polyaniline-carbon nanotube-coated fabric exhibited good electrical conductivity, which decreased slightly after washing in a standard laundry detergent (OMO brand) [33]. Unfortunately, the air permeability of the coated fabric was not investigated in that study either.

The present study proposes a Parylene-C encapsulation strategy to overcome these conflicting requirements. Parylene-C (poly(chloro-p-xylylene)) is a chemically inert, biocompatible, and conformal polymer that has been extensively used as a protective coating in microelectronics, aerospace devices, and biomedical implants [34,35,36,37]. Owing to its exceptional barrier properties, high dielectric strength, and pinhole-free conformal coverage, Parylene-C has long been employed to protect semiconductor circuits, printed circuit boards, and implantable sensors against moisture, ionic contamination, and chemical corrosion. Its biocompatibility and non-cytotoxicity, certified by ISO 10993 standards [38], also make it widely used in neural electrodes, cardiac pacemakers, and drug-delivery microdevices, where direct and long-term contact with biological tissue is required.

In this work, we extend the use of Parylene-C beyond the traditional microdevice scale to textile substrates, which represents an innovative adaptation of this mature coating technology to flexible and porous fibrous materials. Using a chemical vapor deposition (CVD) process, uniform and thickness-controlled Parylene-C films (≈4–16 μm) were conformally deposited on copper-coated polyethylene terephthalate nonwoven fabrics (CuPET). In this CVD process, the dimer is vaporized and pyrolyzed to a monomer, which diffuses into the fibrous network; polymerization proceeds during condensation on the substrate, producing a conformal Parylene-C coating that fully encapsulates the copper-plated filaments without bridging or pore filling. The encapsulation design therefore aims to simultaneously achieve three functional objectives:

(1) Effective isolation of the metallic copper layer from sweat-induced electrolytic corrosion and ion diffusion;
(2) Retention of air and moisture permeability by maintaining partially open porosity within the nonwoven architecture; and
(3) Preservation of electrical continuity and Joule heating capability for reliable active thermal management.

Beyond its excellent chemical stability, the biological safety and non-irritant nature of Parylene-C ensure that the encapsulated fabrics are suitable for direct skin contact applications, eliminating concerns of toxicity or allergic reactions associated with other polymer coatings [39]. The introduction of this biocompatible micro-encapsulation concept into the domain of metalized smart textiles provides a new route toward durable, sweat-resistant, and user-safe electro-thermal materials. The resulting Parylene-C encapsulated CuPET (PyCuPET) fabrics exhibited distinct morphological, functional, and electrochemical advantages compared to uncoated CuPET [40]. SEM and EDS analyses confirmed uniform polymer coverage without cracks or delamination, while thermal gravimetric analysis revealed improved thermal stability and delayed decomposition onset. Electro-thermal testing demonstrated that all PyCuPET samples maintained rapid and uniform heating, reaching 40–45 °C within 2 min under 2 V. When combined with a thermal insulation layer, the equilibrium temperature further increased, confirming the system’s potential for active heating in cold environments. Most importantly, artificial sweat immersion experiments revealed that Parylene-C encapsulation drastically reduced copper ion release, effectively preventing copper dissolution and particle detachment.

Therefore, this study demonstrates a simple yet effective structural solution for balancing metal protection, permeability, and active heating performance in electro-thermal textiles. By combining CVD-based Parylene-C encapsulation with flexible Cu-coated fabrics, the developed composite provides a stable, sweat-resistant, and breathable platform for next-generation self-heating and thermally adaptive wearable systems.

## 2. Materials and Methods

### 2.1. Material

#### 2.1.1. Greige Material

Copper deposition on nonwoven polyester fabrics was previously achieved through the electroless plating technique in our earlier studies [41]; the detailed process and recipe can be checked from Table 1. To obtain materials with improved structural uniformity, in this work a commercially available copper-coated nonwoven fabric, marketed as “MEFTEX,” was procured from Bochemie Ltd. (Bohumin, Czech Republic). Among the product variants, Meftex 20 was selected due to its optimized porosity and planar density, ensuring optimal permeability and surface exposure [42]. Meftex 20 is manufactured via a patented roll-to-roll technological process that combines sequential chemical treatment and electroplating steps. The fabric is constructed from aligned warp PET monofilaments and weft PET monofilaments using a thermal bonding technique. The irregular orientation of certain fibers (i.e., deviation from an ideal net structure) primarily results from minor inaccuracies inherent in industrial-scale fabrication. Due to the presence of thermally bonded junctions, the arrangement and orientation of the fibrous elements are fixed and cannot be altered. The observed structure represents the actual deviation from the theoretical ideal. The electroless copper deposition process shown in Table 1 is cited from our previous work [41] and is provided here only for background reference. In the present study, a commercially available Cu-coated PET fabric (Meftex 20) was used directly, and no metallization process was performed by the authors.

Prior to metallization, surface activation was conducted using CATAPOSIT 44 (Rohm and Haas, Farmsum, Netherlands) at 45 °C for 5 min, following immersion in 10% hydrochloric acid. The pre-activated PET fabric was then introduced into a CuSO_4_-containing bath. Similarly to the electroless plating route, copper nanoparticles were deposited on the fiber surfaces via chemical reduction using a borohydride-based reducing bath. This process resulted in the formation of a continuous, densely packed copper layer across the fabric surface. For simplicity, the copper-coated fabric (Meftex 20) is hereafter referred to as CuPET. The fundamental properties of CuPET are summarized in Table 2.

Another insulation material used was a kind of polyester nonwoven fabric with an acrylic binder purchased from the company SINTEX a.s. (Česká Třebová, Czech Republic) It is always used for thermal insulation due to its high spectral absorbance, which aids in heat preservation. The detailed information of the insulation nonwovens is listed also in Table 2.

All chemicals used in this study were supplied by Sigma-Aldrich (Prague, Czech Republic) and were of analytical grade.

#### 2.1.2. Fabrication of PyCuPET Samples

**Figure 1:**
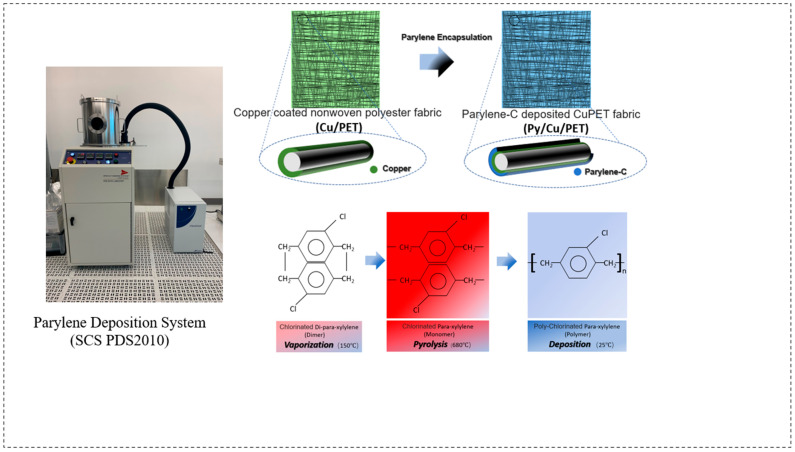
A schematic illustration of the fabrication process. PyCuPET preparation process and sample simulation photo. The insert fiber picture displays the feature of different material layer structures after Parylene encapsulation.

The Parylene-C coating was applied using a CVD process with the Parylene Deposition System (SCS PDS2010, Specialty Coating Systems, Indianapolis, IN, USA) located at CEITEC, Brno, Czech Republic. The CuPET fabric was cut into 13 cm × 13 cm specimens, corresponding to the dimensions of the SCS PDS2010 sample holder. Prior to deposition, those samples were conditioned under ambient laboratory conditions for 24 h and subsequently mounted on a custom paper-based supporting frame to ensure complete and uniform coating coverage. The schematic of the deposition procedure is shown in Figure 1.

The Parylene-C deposition process consisted of three main stages, namely: (1) vaporization of the Parylene-C dimer precursor at 150 °C, (2) pyrolysis of the vaporized dimer into its monomeric form (para-xylene) at 680 °C, and (3) polymerization and deposition of a transparent Parylene-C film onto the substrate under a vacuum pressure of 25 mTorr. The solid Parylene-C dimer served as the raw precursor material, which was thermally cleaved and polymerized in situ during the process. For this study, different precursor masses (2 g, 4 g, 6 g, and 8 g) were used for a single deposition cycle on 13 cm × 13 cm CuPET samples. The corresponding coated samples were denoted as Py2CuPET, Py4CuPET, Py6CuPET, and Py8CuPET, respectively. The sample numbers were designated according to the nominal precursor mass introduced into the CVD system (2–8 g), which was used as the controlled input parameter. The actual deposited polymer mass—which depends on deposition efficiency and process conditions—was experimentally measured and is summarized in Table 3.

The Parylene-C deposition time was automatically determined by the CVD system (SCS PDS2010) according to the loaded precursor mass. Specifically, the process lasted approximately 3 h for 2 g of precursor and up to 10 h for 8 g. The deposition terminated automatically once the precursor was fully consumed, followed by an automatic cooling step. Owing to the gas-phase polymerization mechanism, no unreacted monomer residue remained on the fabric surface.

A notable innovation in this study is the enhanced electrode configuration applied during metallization. Copper foils were attached to both sides of the fabric to improve electrical conductivity and ensure uniform current distribution. Before Parylene-C encapsulation, protective adhesive tape was applied over the copper foils to prevent polymer deposition on the electrode surface. After encapsulation, the tape was removed to expose clean metallic electrodes, enabling efficient current transfer during Joule heating tests. This design ensures stable electrical contact and consistent heating performance while preserving the flexibility of the composite structure.

It should be noted that the mass of Parylene-C deposited on the sample does not directly correspond to the amount of precursor loaded into the deposition chamber, due to chamber volume and deposition efficiency limitations. For instance, with 2 g of precursor, the 13 cm × 13 cm CuPET sample exhibited a mass increase of only 0.02 g after coating. Similar proportional mass increments were observed for the other precursor quantities. The detailed mass changes in the Parylene-C encapsulated sample presented in Table 3

### 2.2. Characterization and Evaluation

#### 2.2.1. Physical Characterization

Surface morphology and elemental composition were analyzed using a scanning electron microscope (SEM, LYRA3, TESCAN, Czech Republic) equipped with an energy-dispersive X-ray spectroscopy (EDS) detector (Bruker, USA). The fabric thickness was measured under a compressive pressure of 1 kPa using a precision thickness gauge (in mm) in accordance with ASTM D5729-97 for nonwoven materials [43]. The thickness of the deposited Parylene-C layer was determined by subtracting the thickness of the uncoated CuPET sample from that of the coated PyCuPET sample. Thermogravimetric analysis (TGA) was performed using a TGA/SDTA851 Thermogravimetric Analyzer (Mettler Toledo, Zurich, Switzerland). The measurements were conducted in accordance with ASTM E1131-20 Standard Test Method for Compositional Analysis by Thermogravimetry and Plastics—Thermogravimetry (TG) of Polymers [44]. Approximately 10 mg of each sample was placed in the sample pan and heated from 25 °C to 600 °C at a constant heating rate of 10 °C/min. All experiments were carried out under a nitrogen atmosphere with a flow rate of 10 mL/min to prevent oxidative degradation.

#### 2.2.2. Air Permeability

Air permeability (AP) measurements were carried out using an FX 3300 air permeability tester (TEXTEST Instruments, Switzerland) at a pressure drop of 200 Pa, following the ISO 9237 standard [45]. Porosity analysis was performed using ImageJ software 1.54p. Due to their ultra-thin structure, both CuPET and PyCuPET samples exhibited high optical porosity. The optical porosity (%) was quantified as the ratio of the total pore area to the total observed area, as determined from the binary-processed SEM micrographs. Five measurements were performed at different positions on each sample, and the average value was reported as the final result.

#### 2.2.3. Water Vapor Permeability Test

Water vapor permeability represents a fabric’s ability to absorb and diffuse water vapor, and it serves as a key indicator of the body’s capacity to maintain thermal balance between heat generation and dissipation through perspiration. According to this research, the water vapor permeability of the fabric was evaluated in accordance with the ISO 11092 standard using a Permetest instrument (Sensora Instruments & Consulting, Liberec, Czech Republic) [46]. Test specimens (13 cm × 13 cm) were conditioned and measured under controlled environmental conditions of approximately 20 °C and 35% relative humidity. During the measurements, each sample was placed on a heated porous plate covered with a water vapor-permeable membrane and exposed to a parallel airflow of 1 m/s. After 2–4 min of operation, the instrument automatically displayed both the relative water vapor permeability and water vapor resistance values. Five measurements were performed at different positions on each sample, and the average value was reported as the final result. A higher water vapor permeability or lower water vapor resistance corresponds to improved moisture transfer and better thermal comfort. Although relative water vapor permeability is not an internationally standardized parameter, it is widely recognized as a practical indicator of fabric breathability and comfort performance.

#### 2.2.4. Thermal Conductivity and Resistance Test

The thermal comfort of fabrics largely depends on their heat transfer characteristics. For this study, the thermal conductivity and thermal resistance of the samples were measured using an Alambeta device (Sensora Instruments & Consulting, Liberec, Czech Republic). The instrument determines heat transfer properties by analyzing transient heat flow through the fabric induced by a temperature difference between the upper (32 °C) and lower (22 °C) measuring plates. During testing, a pressure of 200 Pa was applied between the plates under ambient conditions of approximately 22 °C and 40% relative humidity. Each fabric specimen (≥12 cm × 12 cm, 0.5–10 mm thick) was placed on the lower plate, and heat flux was recorded until thermal equilibrium was reached. The device then automatically calculated and displayed the thermal conductivity and thermal resistance values. All measurements were repeated five times at different sample positions, and the mean values were reported. It is worth noting that the PyCuPET samples are geometrically symmetric across their thickness. During the CVD process, the fabric is freely suspended in the vacuum chamber, allowing Parylene-C to deposit conformally and uniformly on both sides. Therefore, no front–back orientation exists in the thermal measurement.

#### 2.2.5. Joule Heating Test

The Joule heating behavior of the composite sample was examined using an infrared (IR) camera (FLIR E6390, FLIR Systems, Malmö, Sweden) and a regulated DC power supply (TIPA PS3010, TIPA EU, Opava, Czech Republic). The accuracy of this type of camera is ±2 °C. The two opposite edges of each sample were connected to the power supply via electrode clamps spaced 10 cm apart. The IR camera was positioned 20 cm above the sample surface to continuously monitor temperature evolution during the test.

2 V voltages were used to produce heat. The surface temperature at the midpoint between the two electrodes was recorded over a duration of 8 min. For all samples, the surface temperature reached a steady state after approximately 6 min of heating. At this time point, the electrical power was switched off to observe the subsequent cooling (heat dissipation) process. For each sample, 5 times measurements were applied, and the average temperature and 95% confidence interval were calculated to interpret the result significance.

#### 2.2.6. Artificial Sweat Socking and Released Cu Analysis

Artificial sweat was prepared following the formulation recommended by the British Standard BS EN 1811:1999 [47].The solution consisted of 1.3 g/L urea (U5182, Sigma-Aldrich), 10.8 g/L sodium chloride (19015-0350, Junsei, Japan), and 1.2 g/L lactic acid (88%, L6661, Sigma-Aldrich), all dissolved in deionized (DI) water. The pH was adjusted to 6.5 using 0.1 M sodium hydroxide (SDG0-67801, Duksan, Korea).

For the release study, samples (13 cm × 6.5 cm) of the CuPET and PyCuPET were immersed in the artificial sweat solution in an Erlenmeyer flask. Sample mass/solution volume ratio was 1:50 to ensure ion exchange and sufficient infiltration. Then the Erlenmeyer flask was placed on the platform shaker at 90 rpm for 8 h, which simulated the normal working time as the potential for using it as protective clothing in a cold environment. The shaking speed (90 rpm) was selected to approximate the dynamic fabric–body movement during walking (≈90–120 steps/min), ensuring realistic sweat–textile interaction rather than static immersion. This method was referred from the standard EN1811-2023 [48].

After incubation, the textiles were removed, and the remaining solutions were analyzed for metal ion content using inductively coupled plasma mass spectrometry (ICP–MS). The samples were measured using ICP-OES (iCAP-PRO Thermo Fisher Scientific, Waltham, MA, USA), and the measurement error is typically 10%. Nitric acid 66% suprapure was used for acidification. Two types of samples were prepared for copper release analysis. In the first case (referred to as TOTAL Cu), the leachate obtained after immersion in artificial sweat was acidified immediately without filtration and subsequently analyzed. This measurement represents the total copper concentration, including both dissolved ionic copper (Cu^2+^) and undissolved particulate or colloidal copper present in suspension.

In the second case (referred to as dissolved Cu), the leachate was first filtered through a 0.45 µm mixed cellulose ester (MCE) membrane filter to remove suspended solids. The filtrate was then acidified and analyzed, representing only the dissolved Cu^2+^ fraction.

All samples were analyzed using inductively coupled plasma optical emission spectroscopy (ICP–OES, iCAP PRO, Thermo Fisher Scientific), with a typical measurement uncertainty of approximately ±10%. Suprapure 66% nitric acid (HNO_3_, Merck, Darmstadt, Germany) was used for acidification to stabilize metal ions and prevent precipitation prior to analysis.

It should be noted that full immersion was intentionally adopted as a worst-case exposure scenario rather than a direct simulation of practical perspiration-wetting conditions in order to ensure a conservative evaluation of copper ion release under maximum sweat-contact conditions.

## 3. Results and Discussion

### 3.1. Morphology Characterization

**Figure 2:**
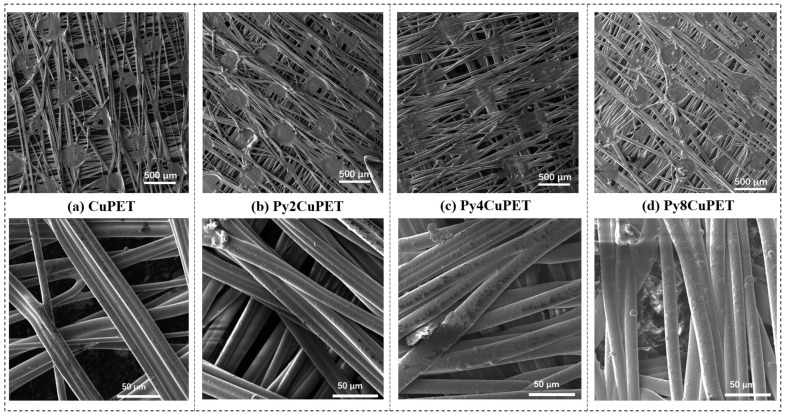

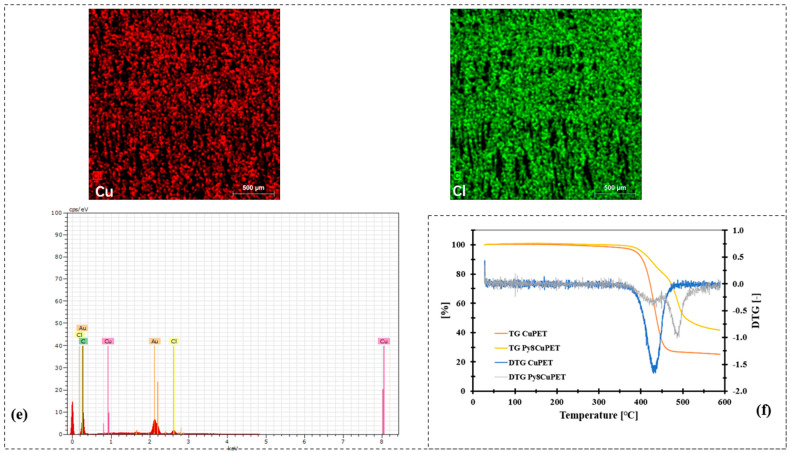
Morphology characterization of CuPET and Parylene-C encapsulated samples (PyCuPET) (**a**) CuPET (**b**) Py2CuPET (**c**) Py4CuPET (**d**) Py8CuPET (**e**) EDS mapping of Cu and Cl, EDS spectrum of the fabric Py8CuPET (**f**) TG and DTG analysis of CuPET and Py8CuPET samples.

The geometrical characterization changes in Parylene-C encapsulated PyCuPET samples are listed in Table 3 and Table 4 and Figure 2.

After the Parylene-C encapsulation process, noticeable morphological and geometrical changes were observed in the Cu-coated nonwoven fabric. The reported coating thickness corresponds to the macroscopic structural thickness increase in the fabric (average of five measurements), rather than a local nanoscale film thickness. This approach is commonly adopted for porous textile substrates, where the coating follows the fiber morphology rather than forming a flat continuous layer. As shown in Table 4, the sample thickness increased progressively from 0.078 ± 0.007 mm (Py2CuPET) to 0.090 ± 0.006 mm (Py8CuPET), indicating the successive accumulation of Parylene-C layers with increasing precursor dosage. Correspondingly, the calculated Parylene-C film thickness increased from 4 µm to 16 µm, confirming the controllable and uniform growth of the polymer coating through the CVD process.

In contrast, both optical porosity and volume porosity exhibited a continuous decrease—from 13.35% to 6.38% and 68.24% to 53.23%, respectively—demonstrating that the deposition of Parylene-C progressively filled the inter-fiber voids and reduced the overall pore volume of the nonwoven structure. This densification effect resulted from the conformal coating of the Parylene-C layer on the fiber surfaces, leading to partial blockage of the fiber junctions and interstitial spaces.

The SEM micrographs (Figure 2a–d) illustrate the distinct morphological transformation of the Cu-coated nonwoven PET fabrics following successive Parylene-C encapsulation. The pristine CuPET sample exhibits clearly defined fibers with an open, interconnected porous structure. The copper layer forms a continuous but rough coating on the fiber surfaces, ensuring good electrical connectivity. After Parylene-C deposition (Py2CuPET–Py8CuPET), the fiber surfaces appear progressively smoother and more compact, as the conformal Parylene-C film uniformly coats both the copper and PET regions. Increasing precursor mass results in a gradual reduction in inter-fiber voids and visible pore spaces, consistent with the decreasing optical and volume porosities reported in Table 4. EDS elemental mapping (Figure 2e) of Py8CuPET supports these observations: the Cu signal remains evenly distributed across the fabric, confirming the integrity of the metallic coating, while a similar even distribution signal of Cl indicates the presence of a carbon-rich Parylene-C overlayer. The absence of cracks or delamination suggests excellent interfacial adhesion between the Parylene-C coating and the CuPET substrate, ensuring coating continuity and flexibility.

The TGA and DTG curves (Figure 2f) further reveal the impact of Parylene-C encapsulation on the thermal behavior of the composite fabrics. The uncoated CuPET exhibits a single prominent degradation step near 400 °C, attributed to PET decomposition. In contrast, the PyCuPET samples display an additional minor weight-loss stage starting in the range of ~450–500 °C, consistent with the high thermal stability of chlorine-substituted Parylene-C, corresponding to the thermal degradation of the Parylene-C layer. Moreover, the main degradation peak of PET shifts slightly toward higher temperatures, implying that the encapsulating polymer acts as an insulating barrier, indicating that the encapsulating polymer acts as a thermal barrier, suppressing heat transfer and delaying degradation even under inert nitrogen conditions. The DTG profiles show a reduced degradation rate and a broader decomposition region for the encapsulated samples, confirming the stabilizing influence of the Parylene-C coating. The slightly higher residual mass of PyCuPET is ascribed to the thermally stable copper content remaining after polymer combustion.

The combined SEM, EDS, and TGA analyses confirm that Parylene-C encapsulation produces a uniform, conformal, and adherent polymer layer that modifies both the morphology and thermal response of the CuPET fabric. The coating effectively seals surface irregularities, reduces porosity, and enhances fiber cohesion without compromising the continuity of the underlying copper network. Thermally, the encapsulated samples demonstrate improved stability and delayed degradation, indicating enhanced heat resistance. These findings highlight that Parylene-C encapsulation not only protects the metallic and polymeric components from environmental and mechanical stress but also enhances the composite’s structural integrity and durability.

**Figure 3:**
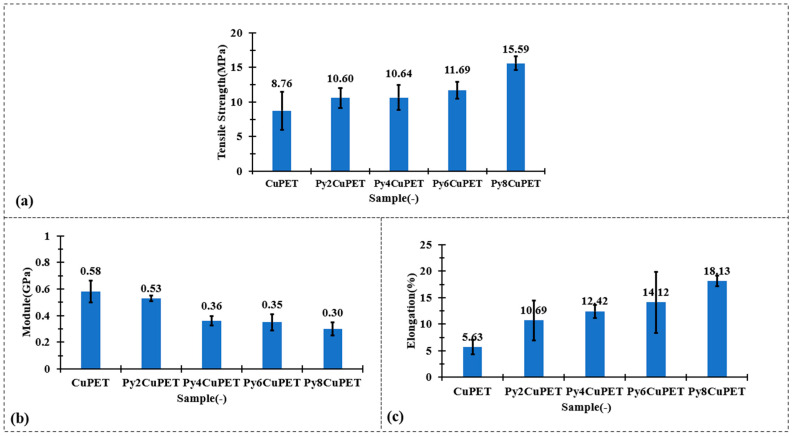
Tensile mechanical performance of CuPET and Parylene-C encapsulated fabrics: (**a**) tensile strength, (**b**) initial modulus, and (**c**) elongation at break.

The mechanical test results in Figure 3 clearly demonstrate that the Parylene-C encapsulation not only preserves but effectively enhances the overall mechanical robustness of the CuPET substrate. Although a gradual decrease in initial tensile modulus is observed after coating—from 0.58 GPa (CuPET) to 0.30 GPa for Py8CuPET—this reduction reflects a shift towards increased structural compliance rather than mechanical degradation. The conformal polymer layer smoothens the fiber–fiber interfaces, promoting controlled inter-fiber slippage at small strains and thus reducing the apparent stiffness in the elastic region. More importantly, both the tensile strength and ultimate elongation exhibit substantial improvement. The tensile strength increases steadily from 8.76 MPa (CuPET) to 15.59 MPa for Py8CuPET, representing an enhancement of nearly 80%. Simultaneously, the elongation at break rises from 5.63% to 18.13%, indicating markedly improved ductility and energy absorption capability. These synergistic trends confirm that the Parylene-C coating reinforces inter-fiber bonding and delays premature fracture while imparting higher resistance to crack initiation and propagation. Consequently, the encapsulated fabrics become stronger yet more deformable, exhibiting a desirable strong–tough–compliant mechanical profile ideally suited for wearable and dynamically deforming electronic textile systems.

Additionally, considering the potential application of PyCuPET fabrics as self-heating inner layers in sandwich-structured technical textiles, their relatively thin and porous architecture offers advantages for integration into protective clothing designed for cold environments. Such structural characteristics can enhance both thermal regulation and wearing comfort while maintaining the material’s flexibility and breathability. The comfort-related properties and further performance evaluations of these composites are discussed in the following sections.

### 3.2. Wearing Comfort-Related Properties of CuPET and PyCuPET

**Figure 4:**
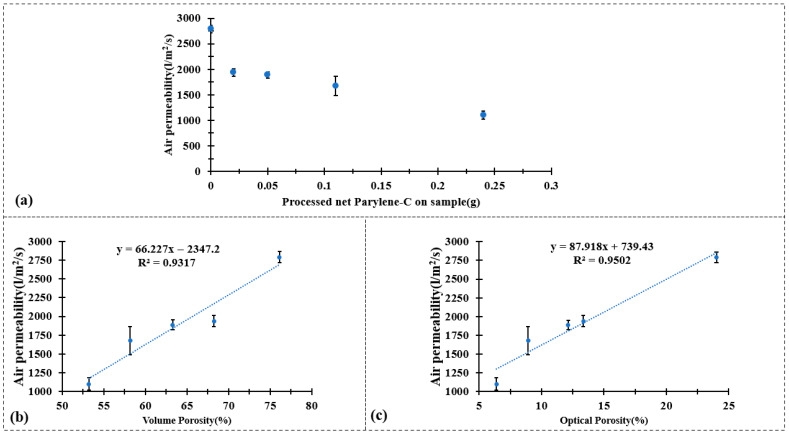
Air permeability test result of (**a**) CuPET and PyCuPET (**b**) The correlation between air permeability and volume porosity and (**c**) The correlation between air permeability and optical porosity.

**Figure 5:**
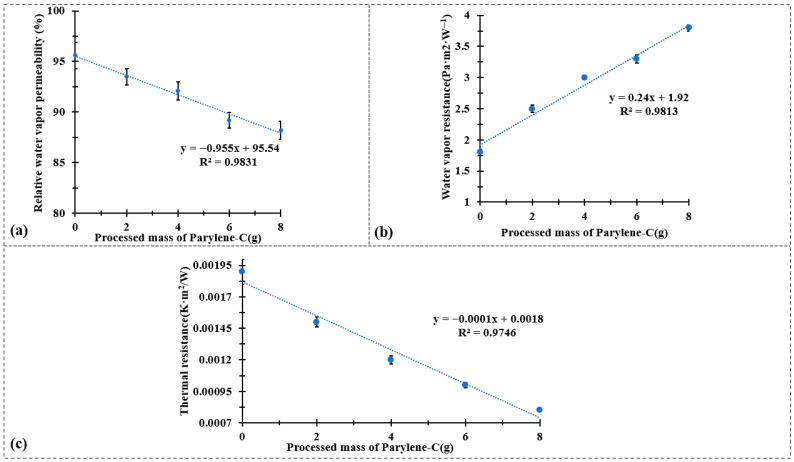
(**a**) Water vapor permeability test results, (**b**) water vapor resistance test results, and (**c**) thermal resistance test results.

Regarding the wearing comfort of textile material, the key performance is normally evaluated from air permeability, water vapor permeability, and thermal properties. These related properties were measured for all samples, and the results are shown in the following Figure 4 and Figure 5.

The air permeability results presented in Figure 4 demonstrate a clear decrease in permeability after the Parylene-C encapsulation process. As shown in Figure 4a, the pristine CuPET sample exhibits the highest air permeability (~3100 L/m^2^/s), which can be attributed to its relatively open fibrous structure and high inter-fiber porosity. After Parylene-C coating, a gradual reduction in air permeability is observed with increasing deposition mass—from Py2CuPET (~1780 L/m^2^/s) to Py8CuPET (~1100 L/m^2^/s). This decline directly reflects the conformal deposition of Parylene-C on the Cu-coated fibers, which partially blocks the pores and narrows the inter-fiber channels, thereby increasing the airflow resistance across the nonwoven structure.

For the Py8CuPET sample, the measured air permeability was 1100.6 L/m^2^/s, which remains comparatively higher than that of conventional clothing fabrics. For reference, typical warp-knitted fleece PET fabrics exhibit air permeability values of 669.2 L/m^2^/s, while lighter warp-knitted underwear fabrics (201 g/m^2^) show values around 900 L/m^2^/s [49]. This indicates that, despite the densification caused by Parylene-C encapsulation, Py8CuPET maintains sufficient air permeability. Therefore, it can be feasibly employed as a functional intermediate layer within multilayer or sandwich-structured technical garments, offering enhanced protective and conductive properties without significantly compromising breathability.

Figure 4b,c further illustrate the strong linear correlation between air permeability and both volume porosity (R^2^ = 0.9317) and optical porosity (R^2^ = 0.9502). These high coefficients of determination indicate that air transport through the composite fabrics is predominantly governed by the geometric porosity of the fibrous network.

The decrease in air permeability after Parylene-C encapsulation mainly originates from the conformal polymer layer increasing the effective fiber diameter and partially narrowing the inter-fiber pores, which leads to a reduction in both optical and volumetric porosity (Table 4). This structural densification naturally increases the airflow resistance and thus causes the observed drop in air permeability.

Figure 5 summarizes the effects of Parylene-C encapsulation on the water vapor and thermal transfer properties of the CuPET composite fabrics. As shown in Figure 5a,b, the relative water vapor permeability decreases linearly with increasing Parylene-C deposition mass (R^2^ = 0.9831), while the water vapor resistance exhibits a corresponding linear increase (R^2^ = 0.9813). This behavior reflects the progressive densification of the fibrous structure and partial blockage of inter-fiber voids by the conformal polymer layer. The deposition of Parylene-C forms a continuous barrier film that hinders water vapor diffusion through the fabric, thereby reducing its overall permeability. Nonetheless, even at the highest coating mass (Py8CuPET), the permeability remains within the acceptable range for technical clothing applications, indicating that the encapsulation process does not excessively compromise comfort-related breathability.

The thermal characteristics, presented in Figure 5c, demonstrate an opposite trend. The thermal resistance decreases almost linearly with the processed mass of Parylene-C (R^2^ = 0.9746). The enhancement in thermal conductivity can be attributed to the intrinsic properties of the Parylene-C coating and its strong interfacial adhesion to the metallic Cu network, which together improve the efficiency of heat transfer through the composite structure. Moreover, the densified fiber network and reduced porosity facilitate more effective heat conduction, as air-filled voids—being thermally insulating—are replaced by the polymer matrix.

These results collectively indicate that Parylene-C encapsulation simultaneously modifies the moisture and heat transport characteristics of CuPET fabrics. The polymer coating introduces a controlled trade-off between vapor permeability and thermal conduction: as the coating mass increases, the material becomes less permeable to moisture but more thermally conductive. This balance is advantageous for self-heating or protective textile systems, where improved heat retention and electrical stability are desired without excessive loss of breathability. In conjunction with the maintained air permeability and enhanced surface uniformity observed in previous sections, these findings confirm that the PyCuPET composites exhibit tunable multifunctional behavior, suitable for advanced applications in wearable heating textiles and thermally adaptive protective garments.

### 3.3. Joule Heating Performance with and Without Thermal Resistance Layer

One of the key reasons to study the conductive textile used as a heat generation layer is due to the Joule heating effect. Joule heating is the process by which an electric current passes through a conductor to generate heat. Due to a continuous copper layer exhibiting a nano-granular surface morphology of CuPET materials, they exhibit the Joule heating effect.

**Figure 6:**
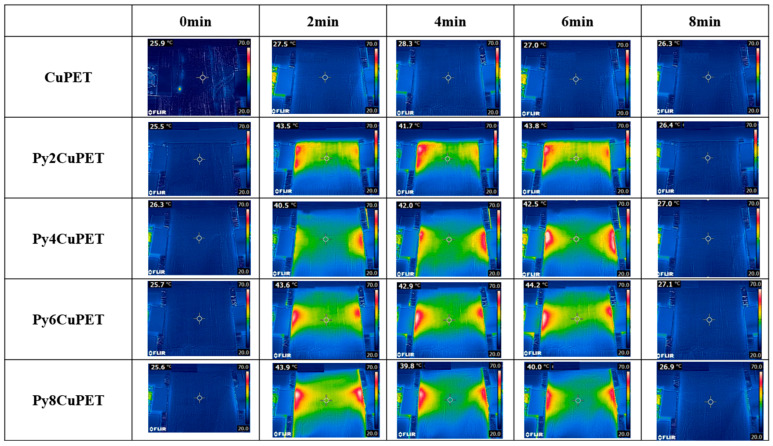
Thermal camera picture of samples during the Joule heating process.

The Joule heating behavior of the CuPET fabrics before and after Parylene-C encapsulation is shown in Figure 6. The infrared thermographic images reveal distinct differences in temperature distribution and heating performance among the sample. The uncoated CuPET sample exhibits less temperature rise under the applied voltage (2 V), indicating an excessive porosity causes the generated heat to dissipate quickly. In contrast, all Parylene-C encapsulated samples (Py2CuPET–Py8CuPET) demonstrate rapid and uniform temperature elevation within the first 2 min of power application, followed by thermal stabilization around 40–45 °C, confirming their effective electrothermal conversion capability.

The Py2CuPET sample reaches approximately 43.5 °C after 2 min, and the temperature remains relatively stable throughout the 8 min heating cycle. Similar behavior is observed for Py4CuPET and Py6CuPET, which show slightly higher steady-state temperatures (up to 44 °C), reflecting enhanced current conduction through the more compact and well-protected Cu network. The Py8CuPET sample exhibits a comparable but slightly lower final temperature, likely due to the thicker Parylene-C layer, which increases interfacial resistance and reduces the overall current density across the conductive paths. The slight drop observed at Py8 suggests that beyond a certain coating thickness, the increasing interfacial resistance may outweigh the benefit of thermal confinement, indicating the presence of an optimum Parylene-C loading. Further refinement with narrower thickness increments will be conducted in future work to precisely locate this optimum. Notably, all encapsulated samples display a homogeneous temperature distribution over the central region, with only minor hot spots near the electrode interfaces, suggesting excellent uniformity of the conductive coating and strong adhesion between the copper layer and the encapsulant.

**Figure 7:**
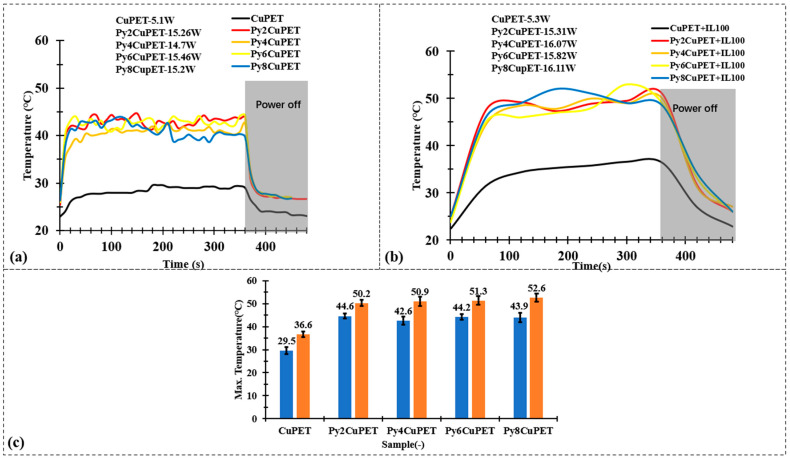
Joule heating test results of (**a**) CuPET and PyCuPET samples and (**b**) CuPET and PyCuPET samples with thermal isolation nonwovens (IL100) on the top. (**c**) The maximum temperature of the Joule heating test.

Figure 7 compares the electrothermal response of CuPET and Parylene-C encapsulated PyCuPET fabrics before and after covering with a nonwoven thermal insulation layer. As shown in Figure 7a, all PyCuPET samples exhibit a rapid temperature increase within the first 60–80 s after power application, reaching a steady-state temperature between 40 and 45 °C, while the uncoated CuPET remains below 30 °C under identical conditions. This demonstrates that the Parylene-C encapsulation enhances the stability and uniformity of the conductive copper network, enabling more efficient Joule heating. The small temperature fluctuations observed during steady state can be attributed to the minor variations in electrical resistance caused by the heterogeneous fiber morphology. When the power supply is switched off (after 6 min), the temperature of all samples decreases quickly, indicating a good heat dissipation capability of the thin and porous fabric structures.

Considering the practical application scenario, the metallized fabrics are intended to serve as the functional middle layer within a sandwich-structured textile system. In this configuration, a thermal insulation layer and an outer protective shell are positioned above the metallized fabric. As demonstrated in our previous studies [42,50], the presence of the thermal insulation layer plays a critical role in determining the overall thermal behavior of such multilayer textile assemblies. Therefore, it is essential to evaluate the electrothermal performance of the metallized fabrics under conditions that include the insulating layer, particularly to assess their heat generation and retention capability in realistic use environments. After applying a nonwoven thermal insulation layer (Figure 7b), the overall heating profiles maintain similar trends, but the steady-state temperatures slightly increase by approximately +6 °C compared with the uncovered condition. This improvement results from the suppression of convective heat loss to the surrounding air, as the insulating layer retains the generated heat near the fabric surface. The PyCuPET samples still display faster heating and higher equilibrium temperatures than CuPET, confirming that the Parylene-C layer does not hinder but rather stabilizes the Joule heating behavior by protecting the metallic network from oxidation and surface discontinuities.

Figure 7c compares the maximum surface temperature reached by CuPET and PyCuPET samples with or without the thermal insulation layer. Under identical input voltage conditions (2 V), the pristine CuPET shows the lowest temperature rise, reaching only 29.5 °C without insulation and 36.6 °C with insulation. In contrast, all Parylene-C encapsulated samples exhibit a statistically significantly higher steady-state temperature, confirming that the conformal coating not only stabilizes the conductive network but also suppresses rapid heat dissipation through the porous structure. With increasing coating thickness, the maximum temperature gradually increases from 44.6 °C (Py2CuPET) to 43.9–44.2 °C (Py6–Py8CuPET) without insulation and further up to 50–52.6 °C when the insulation layer is applied.

These findings suggest that the combination of Parylene-C encapsulation and an external thermal insulation layer provides a synergistic effect, ensuring efficient, uniform, and energy-stable electrothermal performance. Such a configuration allows for controlled heat retention while maintaining structural flexibility—features desirable for wearable thermal management, personal heating textiles, and protective garments designed for cold environments. The PyCuPET composites therefore demonstrate promising adaptability for multilayer smart textile systems where electrical, thermal, and comfort functions must be simultaneously optimized.

### 3.4. Cu Residue After Sweat Socking and Electrical Behavior Change

**Figure 8:**
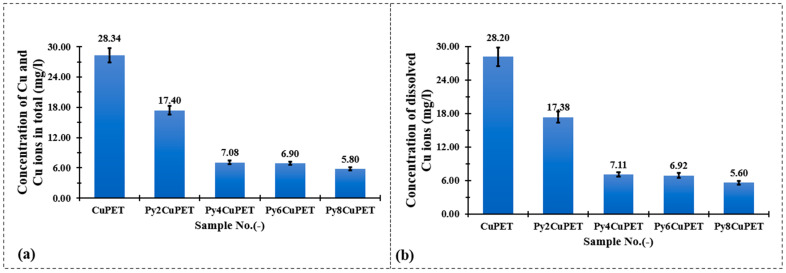
Comparison of total (**a**) and dissolved (**b**) copper ion concentrations measured by ICP-OES after immersion of CuPET and PyCuPET fabrics in artificial sweat.

Figure 8 illustrates the copper release behavior of Cu-coated PET fabrics (CuPET) and their Parylene-C encapsulated counterparts (PyCuPET) after immersion in artificial sweat. Two types of measurements were conducted: (a) the total copper concentration (TOTAL Cu), representing both dissolved and particulate forms of copper, and (b) the dissolved Cu^2+^ concentration, determined after filtration through a 0.45 µm membrane. The results clearly show a significant reduction in copper release after Parylene-C encapsulation.

For the uncoated CuPET, the total released copper reached 28.34 mg/L, with nearly identical dissolved Cu^2+^ content (28.20 mg/L), indicating that the majority of copper was present in ionic form, likely due to the direct exposure of metallic copper to the mildly acidic and chloride-rich artificial sweat. In contrast, the PyCuPET samples exhibited a progressive decline in both total and dissolved copper concentrations with increasing Parylene-C coating mass. The Py2CuPET sample released 17.40 mg/L in total and 17.38 mg/L of dissolved Cu^2+^, while the Py8CuPET sample showed the lowest release, with only 5.80 mg/L (total) and 5.60 mg/L (dissolved). This corresponds to an approximate 80% reduction in copper ion release compared to the uncoated CuPET.

The close similarity between total and dissolved copper concentrations across all samples suggests that most of the released copper existed in ionic form (Cu^2+^) rather than as suspended particulate copper, consistent with the strong chelating ability of lactate and chloride ions in the artificial sweat medium. The substantial decrease in Cu^2+^ concentration with increasing Parylene-C thickness demonstrates the effective barrier function of the polymer coating, which restricts direct contact between the electrolyte and the metallic copper surface. Furthermore, the dense and conformal nature of the Parylene-C film likely minimizes micro-defects and pinholes that could otherwise promote corrosion or ion diffusion.

As expected, the Cu concentrations obtained under full-immersion conditions were higher than the regulatory skin-contact limits defined in EN 1811:2023 [48] (0.5 μg/cm^2^ per week), since the latter is based on perspiration-wetted, non-immersed contact. The present results thus represent the maximum possible release rather than realistic exposure levels during wear.

These findings confirm that Parylene-C encapsulation significantly enhances the chemical stability and corrosion resistance of Cu-coated nonwoven fabrics in perspiration-like environments. The reduced copper ion release not only extends the material’s durability but also mitigates potential cytotoxic or environmental concerns, supporting the safe use of PyCuPET composites in wearable and skin-contact applications.

## 4. Conclusions

A conformal Parylene-C encapsulation strategy was successfully applied to Cu-coated nonwoven PET fabrics via a scalable CVD process, forming uniform and defect-free barrier layers without compromising textile flexibility. The encapsulation effectively stabilized the conductive network, enhancing Joule heating efficiency and heat retention while maintaining acceptable air and moisture permeability for wearable integration. Mechanical robustness was simultaneously improved, with markedly enhanced strength and ductility, indicating better deformation tolerance and long-term durability under bending or handling. The coating provided strong sweat-corrosion protection by significantly suppressing copper ion release, ensuring safety and reliability for skin-contact and perspiration-exposed applications.

Overall, this work demonstrates that Parylene-C encapsulation provides a versatile and scalable solution for developing durable, breathable, and electro-thermally stable conductive textiles. The presented PyCuPET composites combine efficient Joule heating performance, improved safety, and structural integrity, making them promising candidates for smart thermal garments, wearable heaters, and protective clothing for cold environments. Future research will focus on optimizing the encapsulation thickness–conductivity trade-off, long-term cyclic durability under repeated bending and washing, and the integration of sensing or self-regulating heating functionalities to advance next-generation adaptive textile systems.

## Figures and Tables

**Table 1:** Electroless copper deposit method for PET nonwoven fabrics [41].

Process	Chemicals and Processing Parameters	
1. Surface treatment	2.5% NaOH	40 °C for 10 min
2. Activation	1 g/100 mL SnCl_2_	Room temperature for 10 min
	0.05 g/100 mL PdCl_2_	Room temperature for 10 min
3. Deposition	6.5 g/500 mL CuSO_4_·5H_2_O	Sample- Solution ratio: 5 g/500 mL, pH 12.75 (NaOH 2.5 g), 45 °C for 20 min
	10 g/500 mL EDTA·2Na	
	10 g/500 mL KNaC_4_H_4_O_6_·4H_2_O	
	0.04 g/500 mL K_4_[Fe(CN)_6_]·3H_2_O	
	0.005 g/500 m 2′-2′-Bipyridine	
	7.5 mL/500 mL formaldehyde CH_2_O	

**Table 2:** Basic characteristics of copper-coated nonwoven fabrics (CuPET).

Sample	Component	Mass per Unit Area (g/m^2^)	Thickness (mm)	Deposition of Cu per Unit Area (g/m^2^)	Volume Porosity (%)
CuPET (Meftex 20)	Copper-coated PET nonwoven fabric	24.01	0.074 ± 0.008	4.01	76.13
IL100 (Insulation layer)	100% polyester nonwoven with acrylic binder	96.33	5.173 ± 0.4	-	89.12

**Table 3:** Sample mass change after the Parylene-C encapsulation process.

Sample Number	Mass per Unit Area (g/m^2^)	Calculated Parylene-C Mass per Unit Area (g/m^2^)	Net Parylene-C Film Mass in the Size of 13 × 13 cm (g)
Py2CuPET	25.12	1.11	0.02
Py4CuPET	27.22	3.21	0.05
Py6CuPET	30.76	6.75	0.11
Py8CuPET	38.72	14.71	0.24

**Table 4:** Geometrical characterization of PyCuPET series samples.

Sample Number	Sample Thickness (μm)	Optical Porosity (%)	Volume Porosity (%)	Calculated Parylene-C Film Thickness (μm)
Py2CuPET	78	13.35	68.24	4
Py4CuPET	84	12.14	63.29	10
Py6CuPET	88	8.92	58.17	14
Py8CuPET	90	6.38	53.23	16

## Data Availability

The original contributions presented in this study are included in the article. Further inquiries can be directed to the corresponding author.
